# Preparation of Pluronic/Bile salt/Phospholipid Mixed Micelles as Drug Solubility Enhancer and Study the Effect of the PPO Block Size on the Solubility of Pyrene

**Published:** 2014

**Authors:** Li Wang, Min Peng, Yuan Zhu, Shan-shan Tong, Xia Cao, Xi-ming Xu, Jiang-nan Yu

**Affiliations:** a *Department of Pharmaceutics, School of Pharmacy, Jiangsu University, Zhenjiang 212013, P.R. China.*; b *School of Pharmacy, China Pharmaceutical University, Nanjing 210009, P.R. China.*

**Keywords:** Mixed micelles, Solubilization, Hydrophobic, PPO

## Abstract

Pluronic/bile salt/phospholipid mixed micelles (Pluronic/BS/PS-MM) drug carrier system for solubilization hydrophobic drugs was developed. A typical hydrophobic compound, pyrene, was selected as a representative hydrophobic compound to model the hydrophobic drugs. Five Pluronics, F68, F88, F98, F108, and F127 with different PPO chain length were studied. CMC data and solubilization capacities were obtained from a pyrene solubilization method. A closed association model was used to obtain the thermodynamic parameters: Gibbs free energy (ΔG°), enthalpy, (ΔH°) and entropy (ΔS°) of micellization. The results obtained from these experiments suggest that the mixed micelles was more stable and solubilize more pyrene than single one; and the solubilization of pyrene was strong effected by the PPO block size, thus accentuating synergistic interaction mechanism in Pluronic/BS/PS-MM. The study generated an important dataset so as to compare the effect of different Pluronics on solubility capacity of Pluronic/BS/PS-MM.

## Introduction

Solubilization of poorly soluble drugs is one of the most important physicochemical properties for drug development since more than one-third of drugs are poorly water soluble or water insoluble. Numerous drug delivery systems have been studied in an attempt to solubilize drugs and to increase their bioavailability. The techniques generally employed for solubilization of drugs includes micronization, chemical modification, pH adjustment, solid dispersion, complexation, co solvency, hydrotropy, micellar solubilization *etc*. (1-10). The utilization of micelles as drug carriers presents some advantages when compared to other alternatives such as the use of water-soluble polymers and liposomes. Micelles can stay in the body (blood) long enough to provide gradual accumulation in the required area, and their sizes permit them to accumulate in areas with leaky vasculature. Moreover, micelles can be obtained in an easy and reproducible manner in large scale (6). In this context, solubilization is defined as the spontaneous dissolution of a material by reversible interaction with micelles to form a thermodynamically stable isotropic solution that exhibits reduced thermodynamic activity of the solubilized material.

Generally, micelles are divided into two categories: phospholipids mixed micelles and polymeric micelles. Long chain phospholipids form bilayers showing good physiological compatibility, their derived mixed micelles are intensively investigated as drug carriers for many years (7-10). Various therapeutic drugs are commercialized as formulations containing bile salts, phospholipids and/or fatty acids. But the solubilization capacities of these mixed micelles are somewhat lower. Polymeric micelles are made of amphiphilic block copolymers. They are widely researched for drug encapsulation and delivery (11), because the hydrophilic parts could form a water-soluble shell and the inner core created by the aggregation of hydrophobic segments could accommodate hydrophobic drugs and later release them in a controlled way. One of the most famous amphiphilic copolymers used in amphiphilic polymeric micelles is poly(ethylene oxide)-poly(propylene oxide)-poly(ethylene oxide) (PEO-PPO-PEO) block copolymer which is commercially available as Pluronic (12-14). 

The hydrophobic PPO core is capable of loading drugs and the biocompatible hydrophilic PEO corona facilitates long circulation of the micelles in the bloodstream allowing these nontoxic copolymers to be tried in drug dissolution and delivery systems.

The polymeric micelles are still temperature and concentration dependent, although they have higher solubilization capacities compared with phospholipids mixed micelles. Now, mixed surfactants are of great interest in scientific and industrial application. Mixed surfactant systems are often superior in application to that of the individual ones. For example, mixed micelles of ionic and nonionic surfactants show advantageous solubilization behavior and an expanded colloidal stability when compared with the pure nonionic system. The phospholipids mixed micelles have lower critical micelle concentration (CMC) than that of polymeric micelles, so they might overcome the thermodynamic instability present in polymeric micelles. 

To the best of our knowledge, there is no phospholipids-polymeric mixed micelles was reported. Then, it is difficult to understand and predict the solubilization behavior of this mixed surfactant system for solubilization drugs and the selection of the mixed surfactants in this system lacks adequate theoretical basis.

The objectives of this study are (1) to investigate the solubilization of pyrene in Pluronic + bile salt + phospholipid combined mixed micelles and compare with single one, and (2) to investigate the effect of PPO block size of Pluronic on the solubilization of pyrene in this system. The experimental results can be used to understand and predict the solubilization properties of this mixed system and provide valuable information for selection of surfactants on the Pluronic/BS/PS-MM of solubilization drugs. 

## Experimental

*Reagents and standards*

Lecithin was purchased from Shanghai Taiwei Pharmaceutical Industry Co. Ltd. Sodium cholate was purchased from Sinopharm Chemical Reagent Co. Ltd. Pluronics were purchased from Nanjing Well Chemical Co. Ltd. The physicochemical characteristics of the Pluronics used are shown in Table 1. The other chemical reagents were of analytical grade or better.

**Table 1:** Properties of PEO-PPO-PEO triblock copolymers (Pluronics) used in this work.

**Copolymers**	**MW** a	**Average no.of PEO units (x)** b	**Average no.of PPO units (y)** b
F68	8400	152.73	28.97/0.1896
F88	11400	207.27	39.31/0.1896
F98	13000	236.36	44.83/0.1896
F108	14600	265.45	50.34/0.1896
F127	12600	200.45	65.17/0.3251

a The average molecular weights provided by the manufacturer (BASF, Wyandotte, MI).

b The average number of PEO and PPO units were calculated using the average molecular weights.

*Preparation of micelles*

Pluronic/BS/PS-MM were prepared by thin-film evaporation technique as described previously with some modifications (15). In short, sodium cholate and lecithin were dissolved in absolute ethyl alcohol in an ultrasonic bath. Then Pluronic was added and dissolved in the mixture. A lipid film was formed after evaporation of ethyl alcohol by rotary evaporator (Heidolph, Germany). The lipid film was then hydrated and dispersed in 20 mL phosphate buffer (0.01 mol/L, pH 7.4) while stirring at room temperature to form Pluronic/BS/PS-MM. Best ratio of 0.4 g phospholipid, 0.3 g sodium cholate and 0.3 g Pluronic was obtained by phase diagrams analysis. Bile salt/phospholipid mixed micelles (BS/PS-MM) and Pluronic-M were prepared by the same procedure, except Pluronic or sodium cholate/lecithin was not added. The resulting micelles were stored at 4 °C.

*Thermodynamic parameters*

CMC values of the chosen micelles were estimated by the standard pyrene method (16). Briefly, 10 mL of micelle solution was added to tubes containing 20 µL pyrene acetone solution (0.15 mmol/L). The mixtures were incubated for 2 h in an ultrasonic bath followed by 12 h at room temperature with shaking. Free pyrene was removed by filtration through 0.2 μm polycarbonate membranes. The fluorescence of filtered samples was measured at the excitation wavelength of 333 nm using an F-2000 fluorescence spectrometer (Hitachi, Japan). The ratio values of emission intensity at 373 nm and 383 nm (F1/F2) were recorded. The inflection point on the F1/F2 vs. lnC curve, determined by taking the first derivation, represents an lnCMC.

**Table 2:** CMC values of different micelles at different temperatures (10^-5 ^mol/L).

**Temp(** ^o^ **C** **)**	**Pluronic** **-M**					**BS/PS-MM**	**Pluronic/BS/PS-MM**				
	F68	F88	F98	F108	F127		F68	F88	F98	F108	F127
30				54.5	7.14	8.97				1.87	2.37
35			38.5	7.19	2.14	11.21			2.77	2.08	3.32
40		52.6	11.5	2.88	0.64	13.46		3.63	3.23	2.91	4.27
45		21.1	3.23	0.55	0.17	15.70		4.15	3.69	3.32	5.22
50	107	8.42	0.77			17.94	4.06	4.67	4.61		
55	37.5	3.51				20.18	5.41	5.19			
60	10.4					22.43	6.09				
65	2.5					26.91	7.44				

Thermodynamics of micelle formation was well established according to the closed association model. From the thermodynamic point of view, the solubilization can be considered as a normal partitioning of the solute between two phases. The thermodynamic stability ΔG° was calculated by the following equation (6):

Equation (1) $$∆G^{{}^{\circ}}=\mathrm{RT}\mathrm{In}[\mathrm{CMC}]$$

Where R is the gas constant and T is the absolute temperature, ΔG° corresponds to the standard free energy of the pyrene from the solid phase to a mixed micelles system.

The enthalpy of micelle formation can be obtained by applying the Gibbs-Helmholtz equation to equation (1)

Equation (2) $$∆H^{{}^{\circ}}={-\mathrm{RT}}^{2}[\frac{\partial \mathrm{In}[\mathrm{cmc}]}{\partial T}]$$

Once the Gibbs free energy and the enthalpy of micelle formation are obtained, the entropy of micelle formation can be determined by

Equation (3) $$∆S^{{}^{\circ}}=\frac{∆H^{{}^{\circ}}-∆G^{{}^{\circ}}}{T}$$

Based on equations (1), (2) and (3), the Gibbs free energies, the enthalpies, and the entropies of micelles can be calculated. The values are shown in Table 3 and Table 4.

**Table 3:** Gibbs free energies (ΔG°) of different micelles at different temperatures (kJ/mol).

**Temp** **(°C)**	**Pluronic** **-M**					**BS/PS-MM**	**Pluronic/BS/PS-MM**				
	**F68**	**F88**	**F98**	**F108**	**F127**		**F68**	**F88**	**F98**	**F108**	**F127**
30				-18.93	-24.05	-23.48				-27.43	-26.83
35			-20.13	-24.43	-27.53	-23.29			-26.87	-27.61	-26.41
40		-19.65	-23.60	-27.21	-31.12	-23.19		-26.60	-26.91	-27.18	-26.18
45		-22.37	-27.34	-32.02	-35.12	-23.16		-26.68	-26.99	-27.27	-26.07
50	-18.37	-25.19	-31.62			-23.16	-27.15	-26.78	-26.81		
55	-21.51	-27.97				-23.20	-26.79	-26.91			
60	-25.39					-23.26	-26.87				
65	-29.78					-23.10	-26.71				

**Table 4:** Enthalpies (ΔH°) and entropies (ΔS°) of micellization of Pluronic/BS/PS-MM at different temperatures (kJ/mol).

**Temp (°C)**	**ΔH°**					**ΔS°**				
	**F68**	**F88**	**F98**	**F108**	**F127**	**F68**	**F88**	**F98**	**F108**	**F127**
30				-31.52	-39.92				-0.0135	-0.0432
35			-26.26	-32.57	-41.25			0.0020	-0.0161	-0.0482
40		-19.39	-27.12	-33.64	-42.60		0.0231	-0.0007	-0.0206	-0.0525
45		-20.01	-28.00	-34.72	-43.97		0.0210	-0.0032	-0.0235	-0.0563
50	-33.57	-20.65	-28.88			-0.0199	0.0190	-0.0064		
55	-34.62	-21.29				-0.0239	0.0171			
60	-35.68					-0.0264				
65	-36.76					-0.0297				

*Measurement of solubili* *zation capacity*

Excess pyrene was added to the mixed micelles and the mixtures were equilibrated by continuous mixing in a water bath maintained at 32 °C for 72 h. The excess pyrene was removed by centrifugation and the supernatant was suitably diluted for analysis. The solution was measured by UV/VIS spectrophotometer (2401PC, Shimadzu, Japan) at the wavelength of 288 nm and the weight of solubilized pyrene was calculated by calibration curve. To examine the effects of Pluronics on pyrene solubilization, five Pluronic block copolymers were investigated.

## Results and Discussion

*T* *hermodynamic parameters*

**Figure 1:**
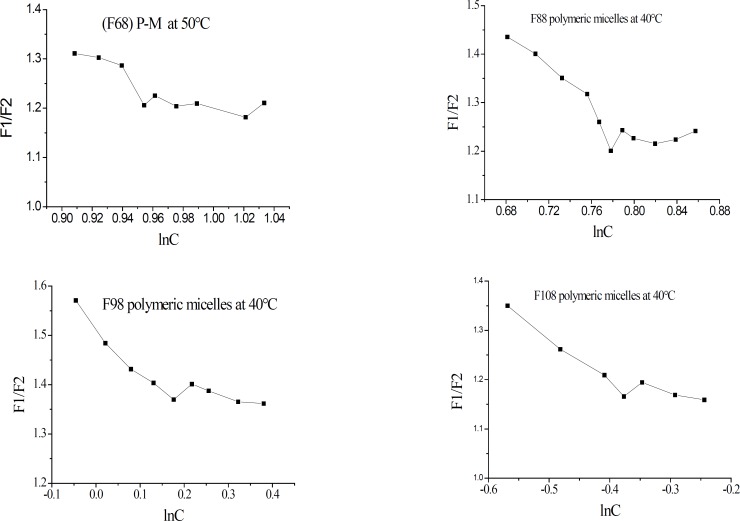
Plots of F1/F2 vs. lnC for different micelle preparations at different temperatures.

In order to estimate the CMC of the studied systems, a series of plots of measured fluorescence intensity ratio (F1/F2) versus lnC of Pluronic in Pluronic/BS/PS-MM, Pluronic-M, and phospholipid in BS/PS-MM were made and are shown in Figure 1. The results of CMC for the different micelles studied are summarized in Table 2.

It has been reported that increasing the PPO block length decreases the CMC, whereas increasing the PEO chain length results in a small increase in the CMC (17). F68, F88, F98, F108 (with low PPO/PEO ratios) are very hydrophilic, PPO units have minimal effects on Pluronic-M CMC from F88 to F108. However, a continuous decrease was obtained for Pluronic/BS/PS-MM CMC with PPO units increasing. This may be due to the possible interactions between the PEO chain with lecithin and sodium cholate. The obviously lower CMC observed for F127 is most likely due to increased instability of the micelles with a larger PPO chain.

It was observed that increasing the temperature led to a decrease in CMC for Pluronic-M. This behavior can be attributed to either less PEO hydration water at the higher temperature or an increase in hydrophobicity of the PPO block, or both according to reference (18). As PEO partly folds around PPO, increasing temperature typically results in expansion of monolayers spread at air/water interfaces, as stronger thermal agitation increases repulsion between the hydrophobic chains (19). Contrary to Pluronic-M, higher CMC for Pluronic/BS/PS-MM was observed at higher temperature. This may be explained as the hydrophobic interaction between PEO and lecithin and sodium cholate (20). As can be seen, the CMC of Pluronic/BS/PS-MM was considerably lower than those of Pluronic-M and BS/PS-MM as a consequence of synergistic interactions between Pluronic copolymer with lecithin and sodium cholate.

The above results are further supported by the variation in the standard Gibbs energy of mixed micelle formation (ΔG°). From the results listed in Table 3, we may conclude that all solubilization process was spontaneous (ΔG°<0). The ΔG° for Pluronic/BS/PS-MM was more negative than those for Pluronic-M and BS/PS-MM, confirming that the solubilization process was energetically more favorable in mixed micellar systems. The values of Pluronic-M decreased with increasing temperature, while Pluronic/BS/PS-MM ΔG° showed little change with increasing temperature meaning that an increase in temperature did not influence the equilibrium toward forms mixed micelles.

Furthermore, the value ΔG° is the sum of the enthalpic (ΔH°) and entropic (-TΔS°) contributions. Regarding the micellization parameters, the contribution of -TΔS° to the Gibbs free energy was much smaller than that of ΔH°, *i.e*., mixed micelle formation was enthalpy-driven. All ΔH° value calculated for Pluronic/BS/PS-MM is negative indicating that the micellization processes of the studied systems were exothermic. This result may be due to the ΔH° values for the transfer of bile salt molecules from an aqueous phase into the phospholipid vesicles was negative. ΔH° was dependent of the number of PPO segment with the exception of F68. General speaking, the hydrophobicity of the copolymer generates more exothermic enthalpies. On the other hand it should be mentioned that the ΔH° tended to become less negative as the temperature was lowered. Such behavior has also been observed in many anionic surfactants system, suggesting that the micellization procedure is salt induced. As the temperature is increased there may be a breakdown in the structure of phospholipid and Pluronic network causing an increase in the energy. This may cause the value of ΔH to fall with increasing temperature.

*S* *olubilization* *capacity*

**Figure 2:**
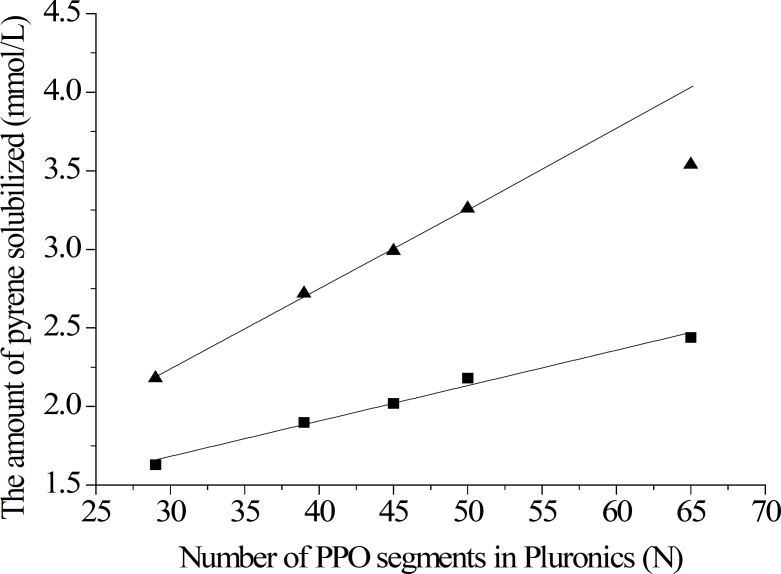
The amount of pyrene solubilized vs. the number of PPO segments (25 °C).

A significant improvement in solubilization of pyrene was seen on the Pluronic/BS/PS-MM compared to Pluronic-M (Figure 2). Interestingly, as the PPO chain length was increased, the amount of pyrene solubilized increased significantly in both Pluronic/BS/PS-MM and Pluronic-M (Figure 2). Good linear correlations were observed. The amount of pyrene solubilized in Pluronic-M vs. PPO segment number was linear (M= 0.0225N + 1.0063, r=0.9942) in all range; the amount of pyrene solubilized in Pluronic/BS/PS-MM vs. PPO segment number was linear (M= 0.0509N + 0.7133, r=0.9998) except the plot at PPO segment number 65. 

(▲) Pluronic/BS/PS-MM, (…) predicted values for Pluronic/BS/PS-MM, (■) Pluronic-M.

Therefore, the amounts of pyrene solubilized in Pluronic-M and Pluronic/BS/PS-MM were strongly correlated with the PPO segment number in Pluronics. Bigger PPO chain led to more pyrene solubilized. It seems reasonable to consider that longer PPO blocks would produce larger hydrophobic inner core, suggesting hydrophobic core of micelles as locus of solubilization. We do observe that this change for Pluronic/BS/PS-MM was much higher than Pluronic-M as shown in Figure 2. This result confirmed that synergism exhibited in Pluronic/BS/PS-MM formation.

## Conclusion

In this study, we have demonstrated that PPO chain length affect the thermodynamic properties of Pluronic/BS/PS-MM micellization which, in turn, affect their solubilization potential for water-insoluble drugs. Good linear correlations between solubilization capacities of Pluronic/BS/PS-MM, Pluronic-M and PPO segments numbers were observed. Pluronic/BS/PS-MM solubilized a significantly higher amount of dye compared to BS/PS-MM and Pluronic-M since synergism exhibited in mixed micelle formation. Further investigations, however, will be required before a more detailed discussion can be done.
